# Memory-related perceptual illusions directly affect physical activity in humans

**DOI:** 10.1371/journal.pone.0216988

**Published:** 2019-05-16

**Authors:** Anne A. Cuperus, Rico T. Disco, Ilja G. Sligte, Milan N. A. van der Kuil, Andrea W. M. Evers, Ineke J. M. van der Ham

**Affiliations:** 1 Health, Medical and Neuropsychology, Leiden University, Leiden, The Netherlands; 2 Brain and Cognition, University of Amsterdam, Amsterdam, The Netherlands; 3 Department of Psychiatry, Leiden University Medical Centre, Leiden, The Netherlands; University of Bath, UNITED KINGDOM

## Abstract

Perceptual illusions help us understand deficits in human perception, but they also have the potential to serve as treatment methods; e.g., to alleviate phantom limb pain. Treatment effects are usually the direct result of a mismatch between false visual feedback and somatosensory/proprioceptive feedback. We aimed to influence physical activity (walking distance) using a memory-related perceptual illusion that relies on a mismatch between a spatially manipulated virtual reality environment and a weakness of memory for a similar, previously experienced environment. Participants' main task was to reproduce a baseline distance three times, by walking on a treadmill while moving through a virtual reality environment. Depending on condition, the environment was either stretched or compressed relative to the previous session, but participants were not informed about these manipulations. Because false, suggestive information can lead to alterations in memory, especially when conveyed through ‘rich’ forms of media such as virtual reality, we expected each manipulation to alter memory for the previous environment(s) and we hypothesized that this would influence walking distance. The results for the first time showed that memory-related perceptual illusions can directly affect physical activity in humans. The effects we found are substantial; stretching previously experienced virtual environments led participants to almost double their initial walking distance, whereas compressing the environments resulted in about half of the initial distance. Possible clinical applications arising from these findings are discussed.

## Introduction

Visual perception was traditionally thought of as a passive, flawless process, in which our eyes function as a perfect camera. However, the study of perceptual illusions demonstrated that it is susceptible to error. Our brain uses other sources of information, such as memory for past events, to *construct* a cognitive understanding of sensory information [1]. What makes this process even more fragile is that our memory itself is not flawless either. That is, a memory becomes labile when reactivated and may be influenced by other cognitive processes, including perception, while in this state [2].

There are obvious downsides to the fallibility of human perception and memory, such as the challenges they present for the justice system, but it can also be used to our benefit. In the nineties, for instance, a mirror visual feedback technique was developed in an attempt to alleviate phantom limb pain [3,4]. It typically involves the use of a mirror across the patient's midline to create the illusion of having two complete limbs [5]. Such a technique has its limitations, because it relies on the presence of an unaffected limb and only allows for symmetric actions. A virtual reality (VR) setup is not necessarily subject to such constraints and may thus provide a better alternative (for a review, see [6]). Seeing a virtual body from a first-person perspective can induce the illusion of ownership over (parts of) this virtual body [7] (cf. the classic ‘rubber hand’ illusion [8]). Moreover, this illusion can still be effective when the virtual body proportions are manipulated, because our body representation is highly plastic; even when a virtual limb triples in length, the illusion may not break [9]. Such false visual body size feedback can further modulate pain [10], but it may also be useful, for instance, in the treatment of patients with anorexia nervosa [11].

VR can be used to present the user with other types of false visual feedback as well, such as the manipulation of perceived walking speed. Normally, the ratio of optic flow to speed of walking, known as the ‘visual gain’, is 1:1. In VR, however, the optic flow needs to be relatively faster for it to appear realistic. Visual gain perception is dependent on several setup-related factors, such as the geometric field of view size [12]. Optimal perceived visual gain was reported to be as low as 1.3:1 [13] and as high as 2:1 [14]. Extremely low ratios (below 1:1) can be used to increase walking speed (but at the expense of realistic perception [15]). VR allows for the manipulation of perceived orientation in a similar fashion. In a technique called ‘redirected walking’, real-world rotations are transformed into increased or decreased rotations in the virtual environment. This allows users to walk through large-scale virtual environments while they physically remain in a small workspace; users can be redirected on a circular arc with a radius of at least 22 m while they believe that they are walking straight [16].

These false visual feedback examples illustrate a clear strength of VR, namely that it is not subject to the limitations of the physical world. What they have in common is that their effects are the direct result of a mismatch between false visual feedback and somatosensory/proprioceptive feedback. In contrast to this, Cuperus et al. tested a perceptual illusion that relies on a mismatch between a manipulated VR environment and a weakness of memory for a similar, previously experienced environment [17]. Participants in their study were patients with intermittent claudication; a cramping pain or discomfort in the legs, which occurs during exercise. They walked on a treadmill while moving through a VR environment three times and were instructed to walk until the pain forced them to stop before each session. All VR sessions contained the same environment, but in the second and third session it was ‘stretched’ and ‘compressed’ (or vice versa) in the direction of its walking trail (by 10% in comparison to the baseline environment). These sessions also included a flag which marked the location of the previously reached walking distance (± 10%, depending on condition), thereby setting visual, attainable goals. None of the participants noticed these manipulations, while they did influence performance; participants walked furthest when interacting with the stretched environment. The authors explained these results in light of the distinction between how we memorize metric and categorical spatial relations (e.g., the side of an object in relation to another object), as proposed by Kosslyn [18]. People are typically not very accurate in memorizing the precise metric properties of objects and their locations, especially after longer temporal delays. In interpreting the environment in the second and third VR sessions, participants were therefore expected to rely mostly on the categorical information they acquired earlier and this information (landmarks and their order) matched with the previous VR session(s).

The study by Cuperus et al. [17] indicated great potential for the use of *memory-related* perceptual illusions to influence clinically relevant physical activity. In the present study, we assessed whether these findings generalize to healthy individuals, because patients with intermittent claudication typically have several comorbid conditions that may affect memory. Furthermore, even if memory was not impacted, participants' walking distance may have been influenced solely by the presence of the virtual flag; i.e., *without* linking the presented visual information to memory. For this reason, and the fact that people normally do not easily reach a pain barrier while they walk on a treadmill, we used a different task in which participants had to reproduce a baseline walking distance. This approach also allowed us to investigate whether the same manipulation can be applied multiple (three) times in a row, with very short time intervals. Because false, suggestive information can lead to alterations in memory [19], especially when conveyed through ‘rich’ forms of media such as VR [20], we expected each manipulation to alter memory for the previous environment(s). We therefore hypothesized that participants in the stretched condition would increase their walking distance each session, whereas participants in the compressed condition would decrease their walking distance each session. Next, in order to explore whether the manipulations also take effect on a basal motoric level, we tested their influence on step length (distance divided by amount of steps). Finally, we made a distinction between participants who may have noticed at least some kind of spatial manipulation during the experiment and participants who did not notice anything at all, and explored whether they behaved differently in terms of walking distance, step length, and a landmark memory task.

We aimed to influence physical activity using a memory-related perceptual illusion that relies on a mismatch between a spatially manipulated VR environment and a weakness of memory for a similar, previously experienced environment. The effects we found are substantial and the findings of our study can be applied in the development of novel clinical applications.

## Materials and methods

### Participants

Participants were recruited via the website proefbunny.nl and social media. Eligible participants were at least 18 years old, and individuals with psychiatric disorders, proneness to motion sickness, a (known) history of heart disease, and/or epilepsy were excluded. A total of forty participants (18 male, 22 female) with a mean age of 26 years (range 18–35; *SD* = 4.1) took part in the experiment. They were randomly distributed over the stretched and compressed conditions.

### Ethical considerations

The study was approved by the Faculty Ethics Review Board of University of Amsterdam's Faculty of Social and Behavioural Sciences (2017-BC-8133), where the study was conducted. The research was carried out in accordance with the provisions of the World Medical Association Declaration of Helsinki [21].

### Tasks and measures

Participants' main task was to reproduce a certain distance three times, by walking on a treadmill while moving through a VR environment. The spatial features of this environment were manipulated during the task; the environment was stretched in the direction of its walking trail by a factor 1.2 relative to the previous session for half of the participants, and for the other half it was compressed by a factor 1.2 relative to the previous session [17], but participants were not informed about these manipulations. Participants were instructed to also play a game (a crystal collection task; see below) while they walked. This dual-task approach was used to mask the actual goal of the experiment, which was to test whether the spatial manipulations influenced walking distance and step length. To check the effectiveness of this masking, we included a questionnaire at the end of the experiment. A number sequence task was used as a distracting filler task between walking sessions. In order to be able to interpret the results of our study within a spatial memory framework, we deemed it important that participants did not deviate in their ability to make accurate estimates of metric properties. A metric estimation task was therefore included and we also added a landmark memory task to assess memory for the categorical information of the VR environment.

#### Walking distance reproduction task

**Fig 1:**
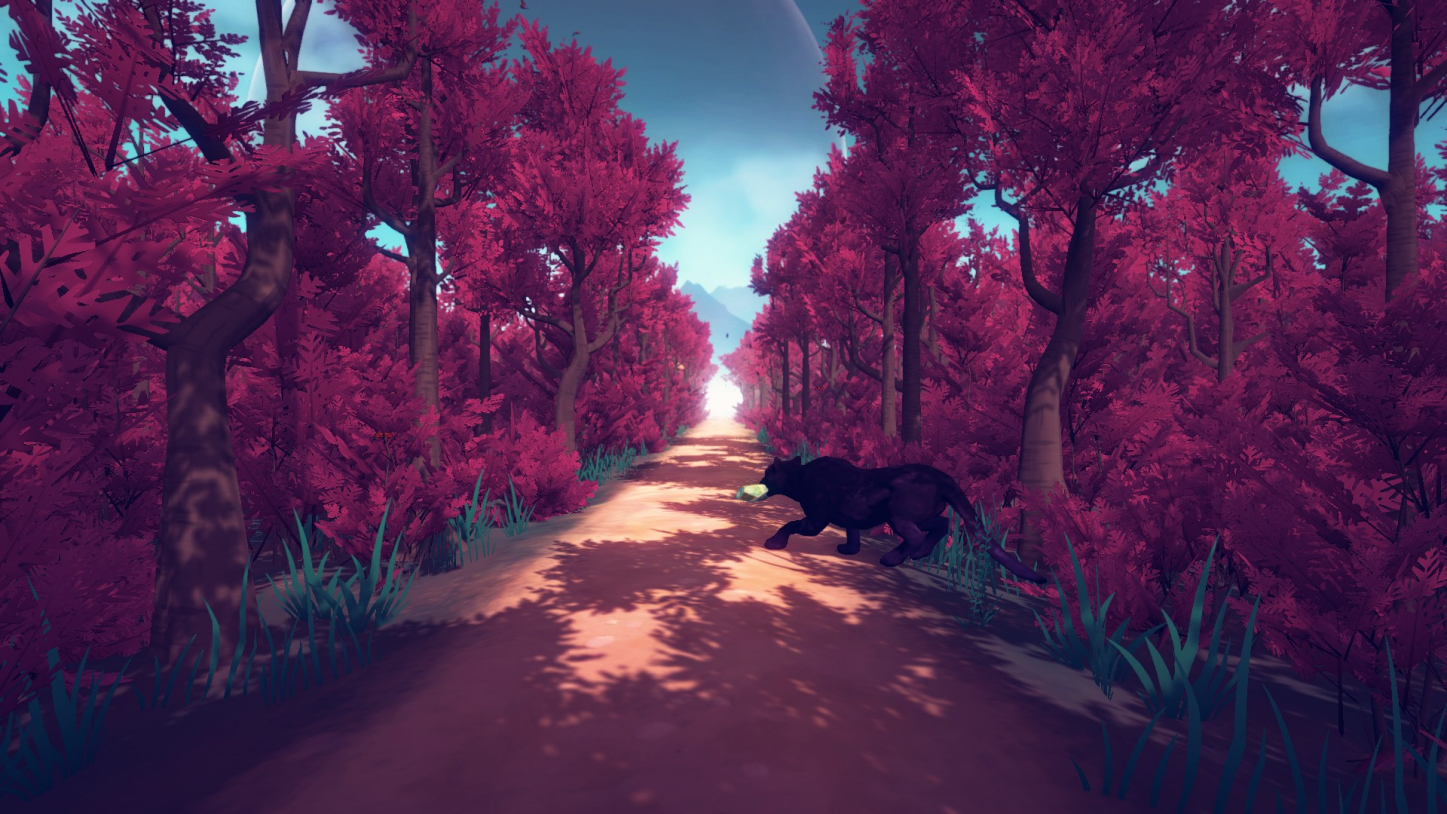
Screenshot of the VR environment; an animal crosses the trail while holding a crystal.

**Fig 2:**
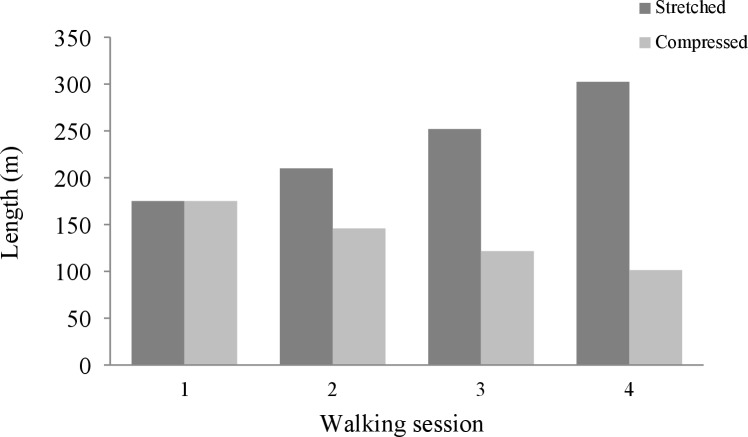
Illustration of how the part of the virtual trail that participants walked in the first (baseline) session stretched or compressed over sessions (factor 1.2).

Participants walked on a treadmill four times while moving over a straight trail in a virtual environment that was presented through a VR headset. This environment consisted of a colourful forest that contained several elements (landmarks) which were encountered in a particular order, such as a pair of giraffes and a pyramid-like structure [17]. In addition to this, the environment contained a fixed amount of crystals (one per 35 m on average) that appeared at varying locations (e.g., in a tree, alongside the trail, or in the mouth of an animal; Fig 1). The entire environment, including its landmarks, was identical for each walking session, apart from its metric properties. That is, depending on condition it was either stretched or compressed by a factor 1.2 relative to the previous session, in the direction of the trail (resulting in stretch/compress factors 1.2, 1.44, and 1.73 compared to the first session; Fig 2). The treadmill was set at the same speed for each session however (3.6 km/h); i.e., the treadmill speed was constant with respect to the lab environment.

Prior to the first walking session, participants were instructed to collect as many crystals while walking as possible before the experimenter would turn off the VR application and the treadmill. These crystals could be collected by being kept in the centre area of the field of vision for 1 s; i.e., without eye tracking. Participants were also asked to pay close attention to the environment, because in the three walking sessions that were to follow their task would not only be to collect crystals again, but also to reproduce the spatial walking distance of the first session (175 m) as accurately as possible. This was done by saying ‘stop’ when they felt this distance was reached, after which the experimenter turned off the VR application and the treadmill. Participants were told they would be awarded a score for both tasks at the end of the experiment.

#### Number sequence task

Participants were presented with 24 sequences of five natural numbers, which all had to be continued by one correct subsequent number. They were instructed to finish as many sequences as possible within 2 min. Solving number sequence tasks is considered a prime example of inductive reasoning, because a problem solver must detect or formulate a relation or rule among elements in a series [22].

#### Landmark memory task

First, participants were asked to mark the elements they remembered crossing during the walking distance reproduction task on a list with descriptions of the six landmarks they actually crossed and of six similar elements that never appeared. One point was awarded for marking a correct element and for not marking an incorrect one (maximum score: 12). Second, participants received printed screenshots of the six landmarks and were asked to place these in the correct order of appearance. One point was awarded for each screenshot that was followed by a screenshot representing a later appearing landmark (maximum score: 5).

#### Metric estimation task

Participants were asked to verbally estimate the dimensions of several geometrically shaped objects (e.g., a cube and a cylinder) and the distances between them (in cm); three times for objects in near (peripersonal) space and three times for objects in far (extrapersonal) space. The objects had a smooth grey texture and were unshaded. Estimates in the peripersonal part of the task were made from a seated position with the objects on a desk in front of participants. Estimates in the extrapersonal part of the task were made from a standing position with the objects on the ground in front of participants (3 m between their feet and the closest object). There was no time limit for the task.

We calculated the absolute difference between each estimate and its related actual size/distance (peripersonal: 22, 9, and 20 cm; extrapersonal: 9, 40, and 100 cm). This difference was divided by the related actual size/distance and then multiplied by 100, resulting in a ‘misestimate percentage’ for each estimate.

#### Questionnaire

The questionnaire contained two open questions: (1) “Did you notice anything during the study and if so, what exactly?” and (2) “What do you think we are investigating?”. Together with any relevant verbal comments during the experiment, these questions were used to make a distinction between participants that did not notice any kind of spatial manipulation and participants who may have noticed at least some kind of spatial manipulation.

### Procedure

After providing written consent, participants carried out the walking distance reproduction task. In order to minimize the risk of falling, they were instructed to hold onto the treadmill's handles during each walking session. Walking distances were read from the treadmill's information display and steps were counted with tally marks. In between walking sessions participants carried out the number sequence task, which allowed the experimenter to set up the VR application with the correct stretch/compress factor for the next session. The last session was followed by the landmark memory task, the metric estimation task, and the questionnaire. Verbal comments indicating that participants noticed any kind of spatial manipulation during the experiment were also written down by the experimenter. Finally, participants were briefed about the actual goal of the study.

### Materials

The VR application was developed in collaboration with Triple (Alkmaar, the Netherlands) and Gamedia (Alkmaar, the Netherlands). The hardware setup consisted of a Focus Fitness Jet 2 fixed speed treadmill (Focus Fitness; Venlo, the Netherlands), an Oculus Rift (first consumer edition; Oculus VR; Menlo Park, California), and a PC equipped with an NVIDIA GeForce GTX 1070 graphics card (NVIDIA; Santa Clara, California). We applied a visual gain of 1.55:1 to our experimental setup, based on a pilot experiment (N = 10) that followed the procedure of Powell, Stevens, Hand, and Simmonds [23]. The statistical analyses were carried out using IBM SPSS Statistics 23 (IBM; Chicago, Illinois).

### Statistical analyses

There was no variance in walking distance for the first walking session, because each participant walked precisely the same distance (baseline); after 175 m was reached, the treadmill was turned off by the experimenter. For the analyses, we therefore calculated the changes in walking distance and step length (distance divided by steps) compared to baseline for each subsequent session. The difference scores were analysed in a mixed ANOVA with walking session as within-subjects factor, and condition and manipulation awareness as between-subjects factors.

## Results

### Sample of participants

Participants' average misestimate percentage was 14.87 (*SD* = 8.94) for the peripersonal part of the metric estimation task and 17.87 (*SD* = 11.86) for the extrapersonal part. One participant in the stretched condition scored outside the range of *M* + 3 *SD* on the peripersonal part of the task and was therefore excluded from the analyses.

### Awareness of manipulation

Based on participants' verbal comments and their responses to the open questions, we concluded that 16 participants (7 male, 9 female) with a mean age of 25.8 years (range 18–35; *SD* = 4.6) may have noticed at least some kind of spatial manipulation; 8 in each condition. These participants reported that they (may have) noticed differences in terms of time, speed, and/or distance between sessions.

With respect to the mixed ANOVA for walking distance, Mauchly's test indicated that the assumption of sphericity was violated (χ^2^(2) = 23.87, *p* < .001); therefore, degrees of freedom were corrected using Greenhouse-Geisser estimates of sphericity (ε = .67). The manipulation awareness × walking session × condition interaction was not significant, *F*(1.33, 46.53) = 3.04, *p* = .054. This interaction was not significant with respect to step length either, *F*(2, 70) < 1.

We carried out two independent samples *t*-tests to find out whether participants who noticed nothing differed from participants who may have noticed some kind of manipulation in their performance on the landmark memory task. However, these revealed no significant differences; neither on the first part of the task (*M* = 11.26, *SD* = .69; *M* = 11.56, *SD* = .51), *t*(37) = -1.49, *p* = .146, nor on the second part of the task (*M* = 4.65, *SD* = .57; *M* = 4.63, *SD* = .62), *t*(37) = .14, *p* = .889.

#### Walking distance

**Fig 3:**
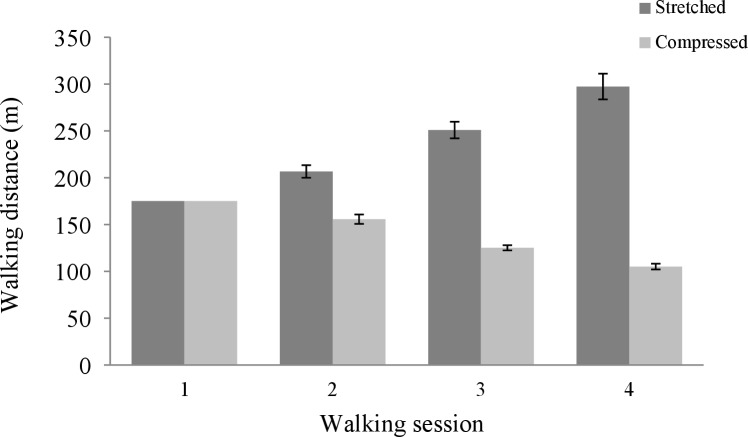
Mean walking distance (m) for each walking session per condition (cf. Fig 2). The error bars represent standard errors.

Fig 3 shows the mean walking distance for each walking session per condition. As predicted, the interaction between condition and walking session was significant, *F*(1.33, 45.19) = 160.41, *p* < .001, η_p_^2^ = .82. Two separate repeated measures ANOVAs were carried out next; one for each condition. For the stretched condition, Mauchly's test indicated that the assumption of sphericity was violated (χ^2^(2) = 18.86, *p* < .001); a Greenhouse-Geisser correction was used (ε = .60). Walking distance significantly differed between walking sessions, *F*(1.20, 21.55) = 75.71, *p* < .001, η_p_^2^ = .81. Post-hoc Bonferroni corrected *t*-tests showed that walking distance significantly increased over sessions, *p* < .001 for each comparison. For the compressed condition, Mauchly's test indicated that the assumption of sphericity was violated as well (χ^2^(2) = 10.70, *p* = .005); a Greenhouse-Geisser correction was used (ε = .69). Again, walking distance significantly differed between walking sessions, *F*(1.38, 26.24) = 139.02, *p* < .001, η_p_^2^ = .88. Post-hoc Bonferroni corrected *t*-tests showed that walking distance significantly decreased over sessions, *p* < .001 for each comparison.

Furthermore, it appears that the distances walked after the baseline session match the scaled virtual distances that are presented in Fig 2 closely; paired samples *t*-tests revealed no significant differences between these distances, *p* > .050 for each comparison.

#### Step length

**Fig 4:**
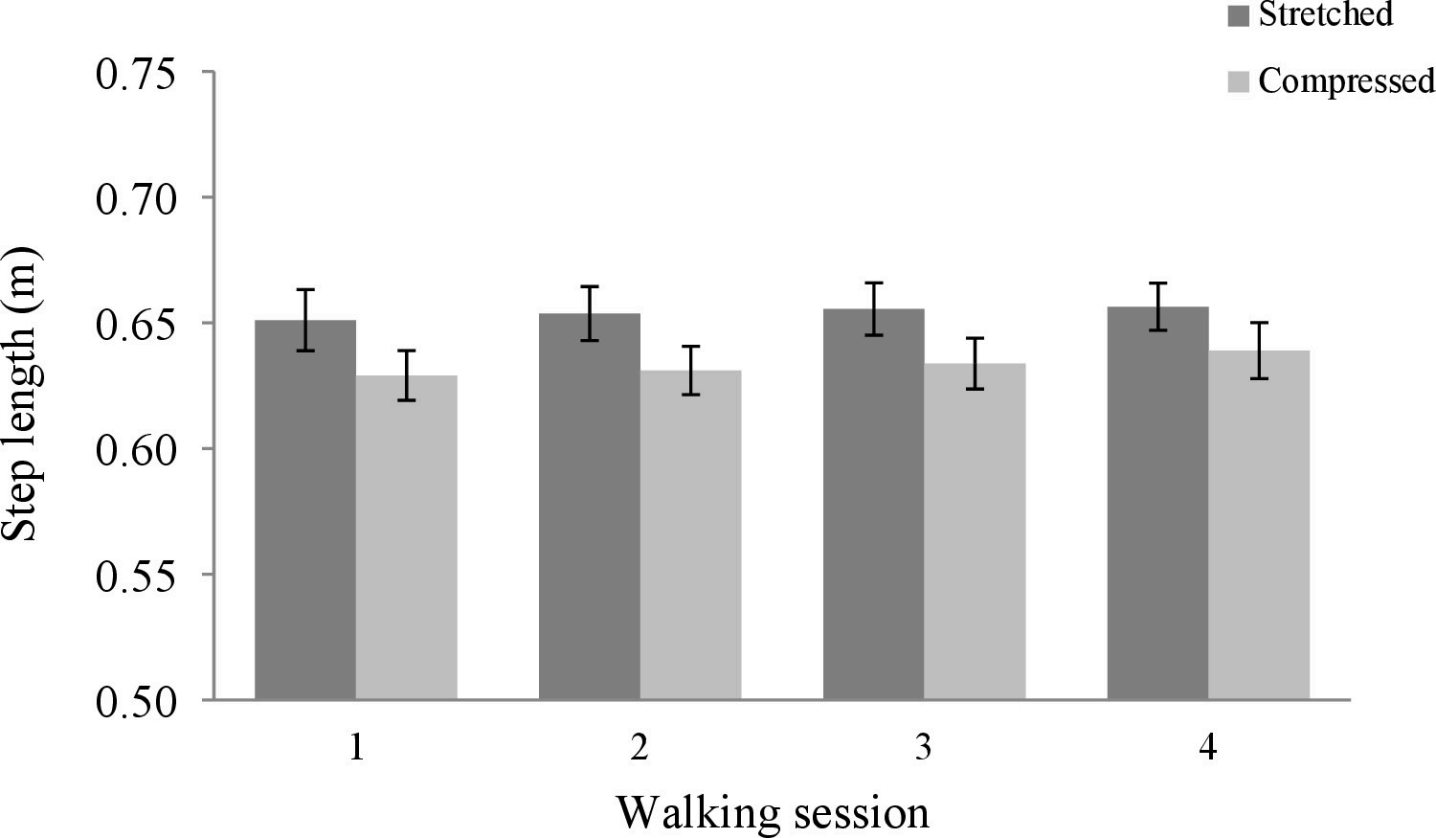
Mean step length (distance divided by steps) for each walking session per condition. The error bars represent standard errors.

Fig 4 shows the mean step length for each walking session per condition. The interaction between condition and walking session on participants' step length was not significant, *F*(2, 70) < 1.

## Discussion

Up till now, the effects of (clinical) applications using perceptual illusions to affect physical activity are the direct result of a mismatch between false visual feedback and somatosensory/proprioceptive feedback. We tested a memory-related perceptual illusion that relies on a mismatch between a spatially manipulated VR environment and a weakness of memory for a similar, previously experienced environment. The results of our study clearly indicate that the effects found by Cuperus et al. were not just a consequence of the fact that their participants consisted of older adults with several comorbid conditions that may affect memory [17]. Moreover, they indicate that the same spatial manipulation (stretching or compressing the VR environment) can effectively be applied multiple times in a row, with very short time intervals. As predicted, participants in the stretched condition increased their walking distance each session, whereas participants in the compressed condition decreased their walking distance each session. The distances walked match the scaled virtual distances almost perfectly. Step length was not influenced by the spatial manipulations, which indicates that the manipulations did not take effect on a basal motoric level.

Although none of the participants were completely aware of the manipulations, sixteen participants did report having the idea that there (maybe) were differences in terms of time, speed, and/or distance between walking sessions. However, it does not seem unlikely that the tasks that *followed* the walking distance reproduction task (the metric estimation task in particular) had a strong influence on retrospective comments and/or answers to the open questions of the questionnaire. More importantly, this group of participants did not perform differently from participants who did not notice anything at all, indicating that their categorical knowledge of the VR environment(s) overruled any suspicions. A limitation of the study that should be noted in this respect is the potential influence of the crystal collection task on walking distance. Although the crystals were hidden in the environment and appeared at varying locations, participants may have used crystal counting as a means of distance estimation. We did not check whether participants used this method, nor did we keep track of the amount of crystals collected.

Future research should look into the further clinical utility of memory-related perceptual illusions combined with walking. In patients with Parkinson's disease, for instance, treadmill training can improve gait [24] and cognitive function [25]. Perhaps the use of the spatial manipulations we used can further increase the effectiveness of treadmill exercise in this population. It might be worth exploring the use a self-paced treadmill instead of a fixed speed treadmill in this regard, because it promotes a more natural gait [26]. Moreover, this would show whether the manipulations can be used to influence walking speed [15]. The utility of memory-related illusions outside the context of walking exercise should also be considered. With respect to reaching tasks for stroke patients, for instance, spatial manipulations might be used to increase maximum reaching distance, thereby enhancing motor recovery [27,28].

The results of our study beg the question what the limits of the perceptual illusions are. Our VR environment was stretched or compressed by a factor 1.2 relative to the previous session [17]; it has to be tested whether similar results are found with a stretch/compress factor 1.5, for instance. Also, it is important to study how many times the same factor can be applied in a row. We expected each manipulation to alter memory for the previous environment(s), but we do not know to what extent memory for the original environment remains intact. Even if previous memories are completely ‘overwritten’ by exposure to manipulated environments, there will still be limits in terms of realism; at some point, one will notice the manipulation because, for instance, an animal alongside the road became thrice as thick. Alternatively, if only the distance between objects in an environment is increased/decreased, this environment may become quite empty/dense at some point. A possible solution to this issue might be to only manipulate the distance between the more obvious landmarks and to leave ‘filler material’ such as trees in place. The effectiveness of such alternatives should also be explored.

In conclusion, the results of our study for the first time showed that memory-related perceptual illusions can directly affect physical activity in humans. The effects we found are substantial; stretching previously experienced VR environments led participants to almost double their initial walking distance, whereas compressing the environments resulted in about half of the initial distance. These findings can be applied in the development of novel clinical applications.
